# Prediction of prognosis in patients with nontraumatic intracranial hemorrhage using blood urea nitrogen-to-creatinine ratio on admission: a retrospective cohort study based on data from the medical information Mart for intensive care-IV database

**DOI:** 10.3389/fneur.2023.1267815

**Published:** 2024-01-05

**Authors:** Peng Chen, YongAn Jiang, JiaHong Cai, Heng Yi Fan, JiaWei Liang, RaoRao Yuan, Hao Wu, YongHong Wang, ShiQi Cheng, Yan Zhang

**Affiliations:** ^1^Department of Neurosurgery, The Second Affiliated Hospital, Jiangxi Medical College, Nanchang University, Nanchang, Jiangxi, China; ^2^Department of Neurosurgery, Shanxi Bethune Hospital, Shanxi Academy of Medical Sciences, Tongji Shanxi Hospital, Third Hospital of Shanxi Medical University, Taiyuan, Shanxi, China

**Keywords:** nontraumatic intracranial hemorrhage, urea nitrogen, creatinine, U-shaped curve, mortality

## Abstract

**Background:**

The blood urea nitrogen-to-creatinine ratio (BUNCR) has been proposed as a potential biomarker for critical illness-induced catabolism. However, its specific relevance and significance in the context of non-traumatic intracranial hemorrhage (NTIH) remains unclear. As such, the primary objective of this study was to determine the role of BUNCR in the prognosis of patients with NTIH.

**Materials and methods:**

All data were sourced from the Medical Information Mart for Intensive Care-IV 2.0 (MIMIC-IV) database. Study outcomes included 30-day and 1-year mortality rates. Univariate and multivariate logistic regression analyses were used to calculate adjusted odds ratio with corresponding 95% confidence interval, and generalized additive model were used to identify both linear and non-linear relationships between BUNCR and mortality rates. A two-piecewise regression model was performed to calculate the saturation effect. Subgroup analyses were performed to evaluate outcome stability in various groups.

**Results:**

A retrospective study of 3,069 patients with NTIH revealed a U-shaped relationship between BUNCR levels and 30-day/1-year mortality. The two-piecewise regression model showed that the inflection points for 30-day and 1-year mortality were 10.455 and 16.25, respectively. On the left side of the inflection point, the 30-day and 1-year mortality rate decreased by 17.7% (OR = 0.823, 95%CI: 0.705–0.960; *p* = 0.013) and 5.3% (OR = 0.947, 95%CI: 0.899–0.999; *p* = 0.046), respectively, per 1 unit increment of BUNCR. On the right side of the inflection point, the 30-day and 1-year mortality rate increased by 1.6% (OR = 1.016, 95%CI: 1.000–1.031; *p* = 0.046) and 3.6% (OR = 1.036, 95%CI:1.019–1.054; *p* < 0.001) per 1 unit decrement of BUNCR. Subgroup analyses revealed consistent results across different strata.

**Conclusion:**

This study identified a nonlinear relationship between BUNCR and mortality in patients with NTIH, indicating that BUNCR may be valuable prognostic marker for early identification and proactive management.

## 1 Introduction

Non-traumatic intracranial hemorrhage (NTIH) refers to a condition in which brain vessel rupture leads to bleeding without any external injury (1), accounting for 10–15% of all stroke cases (2). Although significant improvements have been made in the treatment and management of ischemic stroke, the same does not hold true for NTIH, which continues to exhibit high mortality and morbidity rates. NTIH has a fatality rate exceeding 50% within the first month, with only 20% of survivors achieving complete functional recovery within 6 months (3). The first clinical manifestation of NTIH is the sudden onset of focal neurological deficits, and approximately one-third of patients experience worsening clinical symptoms as the hematoma expands (4). Thus, apart from timely neuroimaging to assess the impact of hematoma(s) on brain function, early evaluation of systemic organ status and disease condition is crucial for improving the prognosis of patients with NTIH.

Blood urea nitrogen (BUN) and creatinine, the ultimate products of nitrogen metabolism in the body, are commonly used indicators of renal glomerular filtration rate and renal function (5, 6). Approximately 95% of creatinine resides within muscle tissues, and its production significantly influenced by muscle mass, rendering it a prevalent hematological marker used in diagnosing muscle depletion disorders (7). However, BUN originates from the breakdown of endogenous protein metabolism and its generation is unaffected by muscle mass. Consequently, the BUN-to-creatinine ratio (BUNCR) is frequently used to detect the presence and assess the degree of renal impairment (8). In previous research, elevated BUNCR has been intricately associated with unfavorable prognoses and heightened mortality rates across a spectrum of medical conditions, encompassing chronic congestive heart failure (CHF) (9), acute decompensated heart failure (10), acute pancreatitis (11), gastrointestinal malignancies (12), ischemic stroke (13), traumatic brain injury (14), and septic shock (15). Intriguingly, heightened BUN levels or BUNCR have also exhibited a correlative surge in inpatient mortality rates among individuals diagnosed with the novel coronavirus disease COVID-19 (16). Based on recent large retrospective studies involving >1,000 trauma intensive care unit (ICU) patients, BUNCR may outperform clinical severity on admission or other patient characteristics in predicting prolonged hospital stay and poor outcomes (17). Similar observations have been made in mixed ICU cohorts (18); thus, BUNCR has been proposed as a potential general biomarker for critical illness-associated catabolism.

The mechanisms underlying brain injury following NTIH can be categorized into primary and secondary injuries. Primary injury is caused by elevated overall pressure due to bleeding and local structural compression (19), whereas secondary injury is induced by pathological responses such as edema, inflammation, and the toxic biochemical and metabolic effects of hematoma components (20). During inflammation, there is a concurrent escalation in protein breakdown metabolism, with the resulting peptides and amino acid byproducts playing a contributory role in irreversible neural damage post-NTIH (21–23). Notably, BUN, a metabolic byproduct, has the potential to increase the risk for delayed cerebral hemorrhage and systemic organ dysfunction in affected patients (24). Considering its association with disease severity in ischemic stroke, inflammation-induced catabolism may play a pivotal role in NTIH. We posit that BUNCR is subject to metabolic regulation in patients with NTIH under the influence of hematoma-driven protein breakdown and inflammatory conditions, subsequently affecting the prognosis of patients with NTIH. Consequently, this ratio, which indirectly reflects the severity of hemorrhage and neural injury, holds promise for assisting in the assessment of survival rates among patients with NTIH.

Given the current research evidence, we collected data from 3,069 patients with NTIH housed in the Medical Information Mart for Intensive Care-IV 2.0 (MIMIC-IV) database, including clinical variables, such as comorbidities and blood biochemical parameters. This study investigated and validated the association between the initial BUNCR on admission and 30-day and 1-year mortality rates in patients with NTIH.

## 2 Materials and methods

### 2.1 Data source

Patient data included in the NTIH study were sourced from the MIMIC-IV database (25), an extensive open-access database at the Beth Israel Deaconess Medical Center (Boston, MA, USA) housing information from 382,278 patients and 524,740 admissions between 2008 and 2019. Essential data, such as patient characteristics, vital signs recorded hourly, comorbid diseases, laboratory investigation results, microbial culture findings, medication records, and survival outcomes, were collected.

The use of the MIMIC-IV database was authorized by the Institutional Review Board of the Beth Israel Deaconess Medical Center and the Massachusetts Institute of Technology (Cambridge, MA, United States). Because the data provided were anonymized, informed consent was not required. To gain access to the database, the necessary online courses and examinations were successfully completed [Record ID: 58572169 (for YAJ)].

### 2.2 Patients and data variables

The data extraction process involved the use of Structured Query Language (SQL) programming in PostgreSQL (version 14.0). The SQL script codes required to extract relevant patient information were sourced from the GitHub website.^1^

Patients diagnosed with NTIH were identified from the MIMIC-IV database according to *International Classification of Diseases* (ICD) codes. Specifically, the Ninth Revision (i.e., ICD-9) codes 430, 431, and 432, and the Tenth Revision (ICD-10) codes I60, I61, and I62. To ensure accuracy, specific exclusion criteria were applied, including patients <18 years of age and those with an ICU stay <24 h. For cases in which patients had multiple ICU admissions, only data from their first admission were considered.

On identifying eligible patients with NTIH, baseline parameter data, recorded immediately after admission to the ICU, were collected. This included demographic information, comorbid diseases, vital signs, laboratory indicators, medications, Glasgow Coma Scale (GCS) scores, and lengths of stay in the ICU and hospital. Vital signs, laboratory indicators, medications, and GCS scores were recorded as initial values within the first 24 h after admission to the ICU.

### 2.3 Clinical outcomes

The clinical endpoints were categorized into two groups: 30-day and 1-year mortality rates. The 30-day mortality rate was assessed in patients who died during hospitalization or within 30-day period following admission to the ICU, while 1-year mortality was defined as death within one year of admission to the ICU.

### 2.4 Statistical analysis

Normality of the continuous variables was examined using the Kolmogorov–Smirnov test. Measurement data conforming to a normal distribution are expressed as mean ± standard deviation (SD), while non-normally distributed data are expressed as median (interquartile range [IQR] [M (Q1, Q3)]). Categorical variables are expressed as composition ratios or frequencies (%). To compare the differences between the two groups, we used chi-square tests or Fisher’s exact test for categorical variables and Student’s t-test or the Mann–Whitney U-test for quantitative variables.

#### 2.4.1 Data analysis

Data analysis was divided into three steps.

*Step 1:* Univariate and multivariate logistic regression analyses were employed to identify variables associated with mortality outcomes in patients with BUNCR. Initially, univariate analysis was performed on relevant variables to determine the covariates for multivariate logistic regression analysis. Through covariate regression analysis, covariates that had an impact on the outcome of patient death beyond 10% were selected, and further adjustments were made based on clinical significance to determine the covariates for multivariate logistic regression (Supplementary Table S1). Thus, three models were established: model 1, without covariate adjustment; model 2, with adjustment for demographic data only; and model 3, with adjustment for covariates, as reported in Supplementary Table S1.

*Step 2:* The nonlinear relationship between BUNCR and patient mortality rate was investigated using a generalized additive model (GAM). If nonlinearity was detected, a recursive algorithm was used to calculate the inflection points and a two-piecewise regression model was constructed on either side of the inflection points. The best-fitting model was determined based on the *value of p* from the likelihood ratio test, with the aim of validating the results using BUNCR as a continuous variable and examining the possibility of nonlinearity.

*Step 3:* Subgroup analysis was performed to explore whether the association between BUNCR and patient mortality rate was influenced by variables such as age, sex, race, chronic kidney disease, hypertension, cerebral infarction, myocardial infarction, diabetes, malignant cancer, and GCS score. The interactions between BUNCR and each variable were tested. All statistical analyses were performed using R version 4.2.0 (http://www.r-project.org; R Foundation for Statistical Computing, Vienna, Austria) and EmpowerStats (http://www.Empowerstat.org/en/; X&Y Solutions Inc., Boston, MA, United States).

## 3 Results

### 3.1 Baseline characteristics of the study cohort

**Figure 1:**
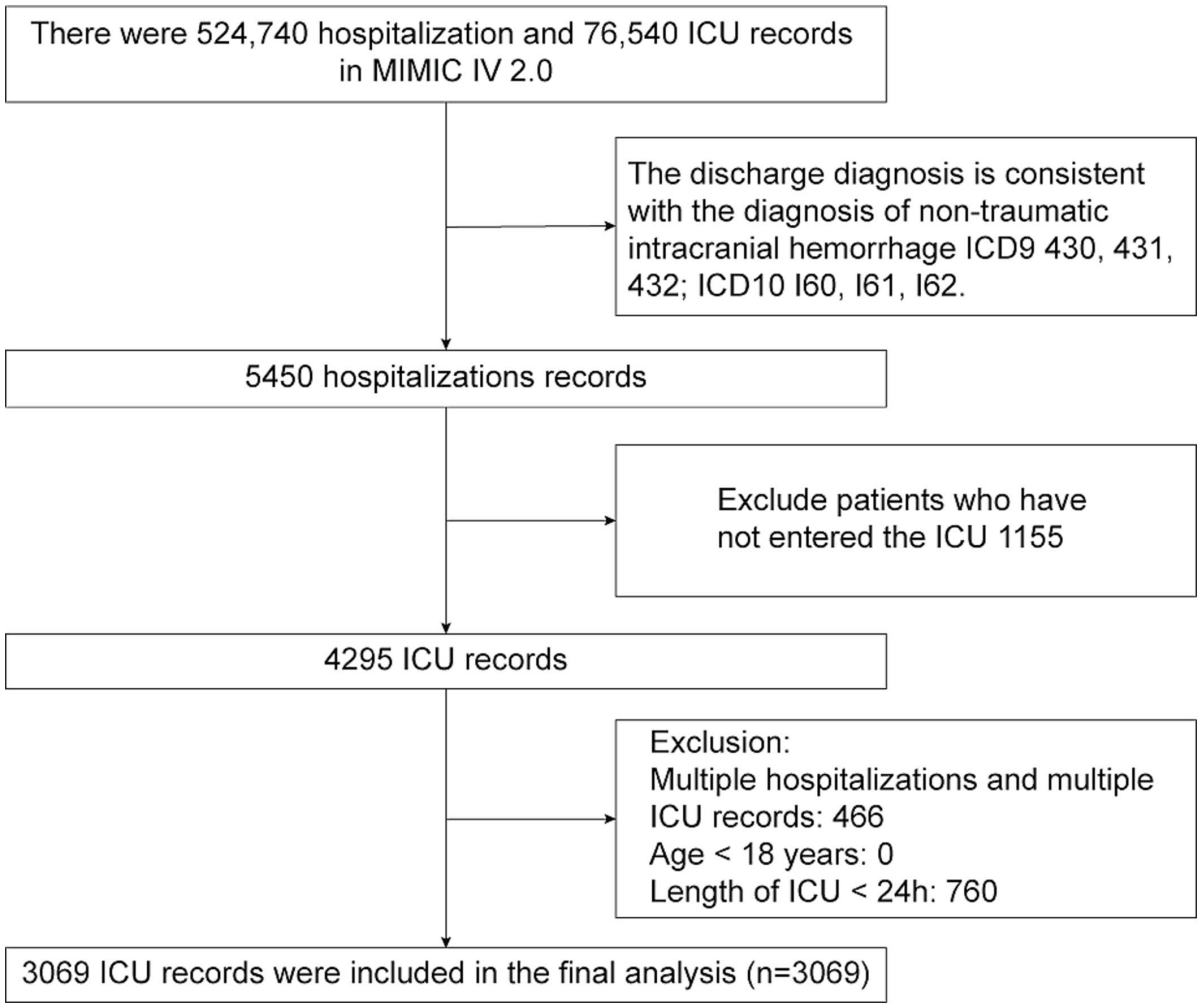
Flow-diagram illustrating patient inclusion in the study.

A comprehensive review of data from 5,450 patients with NTIH housed in the MIMIC-IV database was performed. Following application of the exclusion criteria, 3,069 patients with NTIH admitted to the ICU for the first time were ultimately included in the present study. A flow-diagram illustrating the subject selection process is shown in Figure 1, and baseline characteristics of the subjects in each group are summarized in Table 1. The mean (± SD) age of the 3,069 patients with NTIH was 66.53 ± 15.68 years, comprising 1,429 females (46.56%) and 1,640 males (53.44%). All patients were categorized into 2 groups based on their survival status within 30 days and 1 year (i.e., alive or dead). Statistically significant differences were observed between the 2 groups in terms of age, race, comorbidities (excluding cerebral infarction), vital signs (excluding systolic blood pressure [SBP]), laboratory indicators (excluding lymphocytes and C-reactive protein [CRP]), medications, GCS score, and length of ICU and hospital stay (all *p* < 0.05). Notably, among the 3,069 patients, the BUNCR values recorded were the initial measurements performed within 24 h of ICU admission, with a mean value of 19.07 ± 7.48. A significant difference was observed between the 2 groups (*p* < 0.001).

**Table 1:** Clinical characteristics of ICU patients with nontraumatic intracranial hemorrhage at baseline.

Variables	30-day mortality			1-year mortality		
	Alive group (*n = 2,302*)	Dead group (*n = 767*)	*p*-value	Alive group (*n = 1942*)	Dead group (*n = 1,127*)	*p*-value
Age, (years)	(2302) 64.79 ± 15.61	(767) 71.76 ± 14.72	<0.001	(1942) 63.29 ± 15.47	(1127) 72.13 ± 14.43	<0.001
Male, *n* (%)	1,254 (54.48%)	386 (50.33%)	0.046	1,058 (54.49%)	582 (51.65%)	0.129
**Race, *n* (%)**			<0.001			<0.001
White	1,479 (64.25%)	409 (53.33%)		1,242 (64.00%)	646 (57.32%)	
Black	226 (9.82%)	68 (8.87%)		187 (9.63%)	107 (9.50%)	
Other	312 (13.55%)	78 (10.17%)		273 (14.06%)	117 (10.38%)	
Unknown	285 (12.38%)	212 (27.64%)		240 (12.36%)	257 (22.80%)	
**Comorbid disease, *n* (%)**						
CKD	229 (9.95%)	129 (16.82%)	<0.001	164 (8.45%)	194 (17.21%)	<0.001
Hypertension	1,601 (69.55%)	581 (75.75%)	0.001	1,329 (68.44%)	853 (75.69%)	<0.001
Cerebral infarction	461 (20.03%)	170 (22.16%)	0.204	377 (19.41%)	254 (22.54%)	0.039
Myocardial infarction	412 (17.90%)	177 (23.08%)	0.002	321 (16.53%)	268 (23.78%)	<0.001
Diabetes	482 (20.94%)	215 (28.03%)	<0.001	387 (19.93%)	310 (27.51%)	<0.001
Malignant cancer	192 (8.34%)	74 (9.65%)	0.265	105 (5.41%)	161 (14.29%)	<0.001
CHF	266 (11.56%)	142 (18.51%)	<0.001	194 (9.99%)	214 (18.99%)	<0.001
**Vital signs**						
HR, (times/min)	(2299) 81.00 ± 16.66	(765) 85.12 ± 19.70	<0.001	(1940) 80.79 ± 16.69	(1124) 84.17 ± 18.79	<0.001
RR, (times/min)	(2294) 18.15 ± 4.97	(765) 19.57 ± 5.44	<0.001	(1936) 18.07 ± 4.95	(1123) 19.27 ± 5.34	<0.001
Temperature, (°C)	(2296) 36.82 ± 0.61	(756) 36.73 ± 0.93	0.005	(1937) 36.82 ± 0.61	(1115) 36.76 ± 0.86	0.023
SBP, (mmHg)	(2291) 137.02 ± 22.67	(762) 135.70 ± 26.54	0.183	(1937) 136.66 ± 22.45	(1116) 136.75 ± 25.74	0.919
DBP, (mmHg)	(2291) 74.88 ± 16.82	(762) 72.79 ± 18.09	0.004	(1937) 75.15 ± 16.65	(1116) 73.00 ± 17.95	<0.001
MBP, (mmHg)	(2299) 91.80 ± 17.65	(765) 89.47 ± 18.98	0.002	(1940) 92.03 ± 17.44	(1124) 89.82 ± 18.90	0.001
SpO2, (%)	(2298) 97.54 ± 2.74	(765) 97.84 ± 3.87	0.02	(1939) 97.54 ± 2.70	(1124) 97.75 ± 3.60	0.064
**Laboratory indicators, (Unit)**						
WBC, (x10^9^/L)	(2275) 11.21 ± 11.10	(761) 13.00 ± 10.34	<0.001	(1919) 11.14 ± 9.05	(1117) 12.54 ± 13.55	<0.001
RBC, (x10^12^/L)	(2274) 4.22 ± 0.71	(760) 3.99 ± 0.82	<0.001	(1918) 4.29 ± 0.66	(1116) 3.95 ± 0.83	<0.001
Lymphocytes, (x10^9^/L)	(1463) 1.60 ± 6.86	(482) 1.77 ± 9.08	0.66	(1234) 1.47 ± 1.07	(711) 1.96 ± 12.27	0.164
Monocytes, (x10^9^/L)	(1462) 0.58 ± 0.56	(482) 0.67 ± 0.55	0.002	(1233) 0.56 ± 0.35	(711) 0.68 ± 0.80	<0.001
Neutrophils, (x10^9^/L)	(1462) 8.72 ± 4.66	(482) 10.66 ± 6.10	<0.001	(1233) 8.78 ± 4.62	(711) 9.94 ± 5.82	<0.001
Platelet, (x10^9^/L)	(2274) 224.85 ± 87.17	(762) 204.59 ± 102.65	<0.001	(1918) 226.97 ± 85.24	(1118) 207.42 ± 100.70	<0.001
PT, (sec)	(2216) 13.90 ± 8.52	(749) 15.95 ± 10.93	<0.001	(1868) 13.70 ± 8.13	(1097) 15.64 ± 10.74	<0.001
PTT, (sec)	(2212) 29.83 ± 11.78	(744) 33.29 ± 19.98	<0.001	(1866) 29.73 ± 11.98	(1090) 32.37 ± 17.60	<0.001
INR	(2216) 1.27 ± 0.83	(749) 1.46 ± 1.06	<0.001	(1868) 1.25 ± 0.79	(1097) 1.43 ± 1.04	<0.001
CRP, (mg/L)	(144) 27.66 ± 52.34	(42) 34.05 ± 44.458	0.473	(128) 22.79 ± 44.72	(58) 43.02 ± 59.80	0.011
BUN, (mg/dL)	(2276) 18.57 ± 12.61	(762) 25.14 ± 20.43	<0.001	(1920) 17.49 ± 9.90	(1118) 24.89 ± 20.67	<0.001
Creatinine, (mg/dL)	(2275) 1.07 ± 1.34	(761) 1.33 ± 1.17	<0.001	(1919) 1.02 ± 1.01	(1117) 1.31 ± 1.67	<0.001
BUN / Creatinine Ratio (BUNCR)	(2275) 19.07 ± 7.48	(761) 21.24 ± 10.00	<0.001	(1919) 18.51 ± 6.81	(1117) 21.51 ± 9.95	<0.001
**Medications, *n* (%)**						
Vasopressin	17 (0.74%)	61 (7.95%)	0.601	11 (0.57%)	67 (5.94%)	0.688
Nitrate esters	63 (2.74%)	25 (3.26%)	0.532	52 (2.68%)	36 (3.19%)	0.409
Benzodiazepine	537 (23.33%)	211 (27.51%)	0.111	455 (23.43%)	293 (26.00%)	0.213
β receptor blocker	1,179 (51.22%)	393 (51.24%)	0.564	988 (50.88%)	584 (51.82%)	0.442
Statins	537 (23.33%)	145 (18.90%)	0.604	436 (22.45%)	246 (21.83%)	0.453
Potassium	1,405 (61.03%)	502 (65.45%)	0.55	1,201 (61.84%)	706 (62.64%)	0.443
**GCS, *n* (%)**			<0.001			<0.001
13–15	1,225 (53.22%)	221 (28.89%)		1,092 (56.23%)	354 (31.47%)	
9–12	556 (24.15%)	116 (15.16%)		448 (23.07%)	224 (19.91%)	
3–8	521 (22.63%)	428 (55.95%)		402 (20.70%)	547 (48.62%)	
**Outcome-related measures**						
Length of ICU, (days)	(2302) 7.07 ± 7.64	(767) 5.25 ± 4.88	<0.001	(1942) 6.81 ± 7.10	(1127) 6.29 ± 7.07	0.049
Length of hospital, (days)	(2302) 14.33 ± 14.44	(767) 7.35 ± 6.52	<0.001	(1942) 13.31 ± 13.19	(1127) 11.35 ± 13.32	<0.001

ICU, intensive Care Unit; CKD, chronic kidney disease; CHF, congestive heart failure; HR, heart rate; RR, respiratory rate; SBP, systolic blood pressures; DBP, diastolic blood pressures; MBP, mean arterial pressures; SpO2, oxygen saturation; WBC, white blood cell; RBC, red blood cell; PT, prothrombin time; PTT, partial thromboplastin time; INR, international normalized ratio; CRP, C-reactive protein; BUN, blood urea nitrogen; GCS, Glasgow coma scale.

In Table 1, GCS scores were categorized for 765 cases in the ‘dead group’ for both 30-day and 1-year mortality. Two cases in the ‘dead group’ for 30-day mortality and 1-year mortality had missing GCS scores, accounting for the discrepancy in the total number of cases.

### 3.2 Univariate analysis

Univariate analysis indicated that several factors were associated with increased 30-day and 1-year mortality risk (Table 2), including age, comorbidities (such as chronic kidney disease, hypertension, myocardial infarction, diabetes, malignant cancer, and CHF), vital signs (such as heart rate and respiratory rate), and various laboratory indicators (including BUN, creatinine, BUNCR, white blood cell count, monocytes, neutrophils, prothrombin time, partial thromboplastin time, international normalized ratio (INR), and GCS (score, 3–8)) (odds ratio [OR] >1; *p* < 0.05). Other factors, including sex, race, cerebral infarction, SBP, oxygen saturation (SpO_2_), lymphocytes, CRP, and medications, were found not to be significantly associated with 30-day or 1-year mortality (*p* > 0.05).

**Table 2:** Univariable analysis for associations of BUNCR with 30-day mortality and 1-year mortality in ICU patients with nontraumatic intracranial hemorrhage.

Variables	30-day mortality		1-year mortality	
	OR 95%CI	*p*-value	OR 95%CI	*p*-value
Age	1.032 (1.026, 1.038)	<0.00001	1.041 (1.035, 1.047)	<0.00001
**Gender**				
Male	Ref		Ref	
Female	1.181 (1.003, 1.391)	0.04618	1.121 (0.967, 1.298)	0.12868
**Race**				
White	Ref		Ref	
Black	1.088 (0.812, 1.458)	0.57163	1.100 (0.852, 1.421)	0.46498
Other	0.904 (0.689, 1.186)	0.46594	0.824 (0.650, 1.044)	0.10862
Unknown	2.690 (2.183, 3.314)	<0.00001	2.059 (1.686, 2.515)	<0.00001
**Comorbid disease**				
CKD	1.830 (1.449, 2.311)	<0.00001	2.254 (1.805, 2.816)	<0.00001
Hypertension	1.368 (1.134, 1.650)	0.00106	1.436 (1.216, 1.696)	0.00002
Cerebral infarction	1.137 (0.932, 1.387)	0.20463	1.208 (1.009, 1.445)	0.0391
Myocardial infarction	1.376 (1.128, 1.679)	0.00165	1.576 (1.313, 1.890)	<0.00001
Diabetes	1.471 (1.220, 1.773)	0.00005	1.525 (1.284, 1.810)	<0.00001
Malignant cancer	1.173 (0.886, 1.555)	0.26545	2.916 (2.253, 3.774)	<0.00001
CHF	1.739 (1.392, 2.172)	<0.00001	2.112 (1.712, 2.606)	<0.00001
**Vital signs**				
HR	1.013 (1.008, 1.018)	<0.00001	1.011 (1.007, 1.015)	<0.00001
RR	1.053 (1.037, 1.069)	<0.00001	1.046 (1.031, 1.061)	<0.00001
Temperature	0.848 (0.756, 0.951)	0.00497	0.886 (0.798, 0.984)	0.02358
SBP	0.998 (0.994, 1.001)	0.18305	1.000 (0.997, 1.003)	0.91908
DBP	0.993 (0.988, 0.998)	0.00372	0.993 (0.988, 0.997)	0.00093
MBP	0.993 (0.988, 0.997)	0.00196	0.993 (0.989, 0.997)	0.00111
SpO2	1.037 (1.006, 1.068)	0.01917	1.024 (0.998, 1.051)	0.06536
**Laboratory indicators**				
WBC	1.017 (1.005, 1.028)	0.00374	1.017 (1.005, 1.028)	0.00461
RBC	0.661 (0.592, 0.739)	<0.00001	0.521 (0.468, 0.579)	<0.00001
Lymphocytes	1.003 (0.990, 1.015)	0.66478	1.010 (0.993, 1.026)	0.24828
Monocytes	1.308 (1.078, 1.587)	0.00656	1.583 (1.273, 1.967)	0.00003
Neutrophils	1.071 (1.050, 1.093)	<0.00001	1.044 (1.026, 1.064)	<0.00001
Platelet	0.997 (0.996, 0.998)	<0.00001	0.998 (0.997, 0.998)	<0.00001
PT	1.021 (1.012, 1.031)	<0.00001	1.026 (1.016, 1.037)	<0.00001
PTT	1.014 (1.009, 1.020)	<0.00001	1.013 (1.007, 1.018)	<0.00001
INR	1.235 (1.125, 1.357)	<0.00001	1.296 (1.164, 1.444)	<0.00001
CRP	1.002 (0.996, 1.009)	0.47333	1.007 (1.001, 1.014)	0.01708
BUN	1.028 (1.022, 1.034)	<0.00001	1.043 (1.036, 1.051)	<0.00001
Creatinine	1.176 (1.093, 1.264)	0.00001	1.275 (1.174, 1.385)	<0.00001
BUN / Creatinine Ratio (BUNCR)	1.030 (1.020, 1.040)	<0.00001	1.046 (1.036, 1.056)	<0.00001
**GCS**				
13–15	Ref		Ref	
9–12	1.156 (0.904, 1.479)	0.24690	1.542 (1.262, 1.884)	0.00002
3–8	4.554 (3.758, 5.517)	<0.00001	4.197 (3.520, 5.005)	<0.00001

Blood urea nitrogen-creatinine ratio, BUNCR; ICU, intensive Care Unit; OR, odds ratio; CI, confidence interval; CKD, chronic kidney disease; CHF, congestive heart failure; HR, heart rate; RR, respiratory rate; SBP, systolic blood pressures; DBP, diastolic blood pressures; MBP, mean arterial pressures; SpO2, Oxygen saturation; WBC, white blood cell; RBC, red blood cell; PT, prothrombin time; PTT, partial thromboplastin time; INR, international normalized ratio; CRP, C-reactive protein; BUN, blood urea nitrogen; GCS, Glasgow coma scale.

### 3.3 Analysis of non-linear relationship

**Figure 2:**
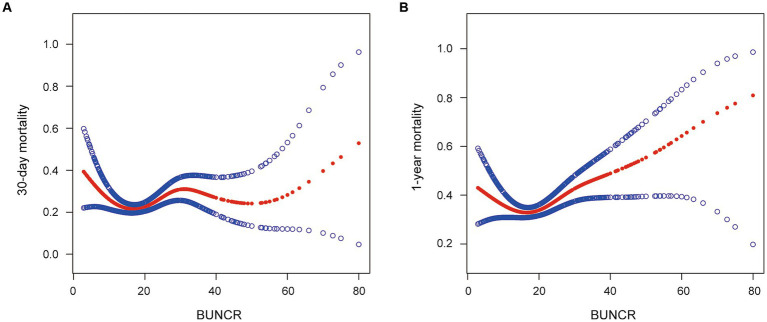
**(A, B)** Smoothed curves for the relationships between blood urea nitrogen-to-creatinine ratio (BUNCR) and mortality risk in patients with nontraumatic intracranial hemorrhage.

As shown in the smoothing spline, BUNCR demonstrated a non-linear relationship with patient mortality (Figure 2). After full adjustment for covariates (sex, age, race, chronic kidney disease [CKD], hypertension, cerebral infarction, myocardial infarction, diabetes, malignant cancer, CHF, GCS, heart rate, respiratory rate, temperature, SBP, SpO_2_, lymphocytes, monocytes, neutrophils, WBC count, red blood cell count, platelet count, and INR), the two-piecewise regression model revealed inflection points of 10.455 and 16.25 for the 30-day and 1-year mortality rates, based on BUNCR (Table 3). Subsequently, OR values for NTIH mortality on either side of the inflection point were computed using the fitted logistic regression model. On the left side of the inflection point, the incidence of 30-day mortality decreased 17.7% per 1 unit in BUNCR (95% CI 0.705–0.960; *p* = 0.013). On the right side of the inflection point, the incidence of 30-day mortality increased 1.6% per 1 unit in BUNCR (95% CI 1.000–1.031; *p* = 0.046). On the left and right sides of the inflection point, the 1-year mortality decreased and increased 5.3% (95% CI 0.899–0.999; *p* = 0.046) and 3.6% (95% CI 1.019–1.054; *p* < 0.001) per 1 unit in BUNCR, respectively.

**Table 3:** Two-piecewise regression model to evaluate relationship between BUNCR with 30-days mortality and 1-year mortality.

	30-day mortality		1-year mortality	
	BUNCR (OR 95% CI)	*P*-value	BUNCR (OR 95% CI)	*p*-value
Fitting model by one-line regression	1.009 (0.995, 1.024)	0.2223	1.022 (1.008, 1.036)	0.0022
Fitting model by two-piecewise regression				
The Inflection Point of BUNCR	10.455		16.25	
< Inflection Point	0.823 (0.705, 0.960)	0.0131	0.947 (0.899, 0.999)	0.0460
> Inflection Point	1.016 (1.000, 1.031)	0.0461	1.036 (1.019, 1.054)	<0.0001
*P* for log likelihood ratio test		0.011		0.004

Blood urea nitrogen-creatinine ratio, BUNCR; OR, odds ratio; CI, confidence interval.

Adjusted: age, gender, race, chronic kidney disease, hypertension, cerebral infarction, myocardial infarction, diabetes, malignant cancer, congestive heart failure, Glasgow coma scale, heart rate, respiratory rate, temperature, systolic blood pressures, Oxygen saturation, lymphocytes, monocytes, neutrophils, white blood cell, red blood cell, platelet, international normalized ratio.

### 3.4 Association between BUNCR and patient prognosis

Three models were constructed to explore the association between BUNCR and patient mortality (Table 4). In crude model adjusted for none, when BUNCR ≤10.455 and ≤ 16.25, BUNCR was associated with a reduced risk of 30-day mortality (OR = 0.826 [95% CI 0.706–0.967]; *p* = 0.017) and 1-year mortality (OR = 0.955 [95% CI 0.912–0.999]; *p* = 0.044), respectively. After adjustment for different confounders, the association between BUNCR and patient mortality remained significant in both adjusted model 1 and model 2 when BUNCR was ≤10.455 and ≤ 16.25 (OR < 1). In the above models, when BUNCR was >10.455 and > 16.25, it was associated with increased risk of 30-day mortality and 1-year mortality, respectively (OR > 1).

**Table 4:** Association between BUNCR and 30-day mortality and 1-year mortality in different models.

Variable	Crude model		Adjusted model I		Adjusted model II	
	OR (95%CI)	*p*-value	OR (95%CI)	*p*-value	OR (95%CI)	*p*-value
30-days mortality						
BUNCR ≤10.455	0.826 (0.706, 0.967)	0.01717	0.849 (0.724, 0.996)	0.04438	0.962 (0.637, 1.452)	0.8525
BUNCR >10.455	1.037 (1.026, 1.048)	<0.00001	1.028 (1.018, 1.039)	<0.00001	1.015 (1.000, 1.031)	0.05517
1-year mortality						
BUNCR ≤16.25	0.955 (0.912, 0.999)	0.04464	0.919 (0.876, 0.963)	0.00048	0.958 (0.888, 1.033)	0.26401
BUNCR >16.25	1.057 (1.043, 1.072)	<0.00001	1.052 (1.038, 1.067)	<0.00001	1.036 (1.017, 1.055)	0.00022

Blood urea nitrogen-creatinine ratio, BUNCR; OR, odds ratio; CI, confidence interval.

Crude model: did not adjust covariates.

Adjusted model I: age, gender.

Adjusted model II: age, gender, race, chronic kidney disease, hypertension, cerebral infarction, myocardial infarction, diabetes, malignant cancer, congestive heart failure, Glasgow coma scale, heart rate, respiratory rate, temperature, systolic blood pressures, Oxygen saturation, lymphocytes, monocytes, neutrophils, white blood cell, red blood cell, platelet, international normalized ratio.

### 3.5 Results of subgroup analysis

Subgroup analyses were performed to investigate the stability of the association between BUNCR levels and 1-year mortality (Table 5). The associations were similar in every stratum and no interactions were observed (*p* > 0.05).

**Table 5:** Adjusted association of BUNCR with 1-year mortality in prespecified and exploratory subgroups.

**Variables**	**BUNCR ≤ 16.25**			**BUNCR > 16.25**		
	**OR (95%CI)**	***P*-value**	***P* for interaction**	**OR (95%CI)**	***P*-value**	***P* for interaction**
**Age**						
≤ 68	0.956 (0.897, 1.020)	0.1717	0.712	1.057 (1.034, 1.080)	<0.0001	0.4389
> 68	0.975 (0.898, 1.059)	0.5432		1.045 (1.026, 1.065)	<0.0001	
**Gender**						
Female	0.983 (0.907, 1.065)	0.6687	0.5212	1.046 (1.027, 1.065)	<0.0001	0.4468
Male	0.951 (0.891, 1.014)	0.127		1.058 (1.033, 1.083)	<0.0001	
**Race**						
White	0.909 (0.845, 0.979)	0.0112	0.1333	1.049 (1.031, 1.067)	<0.0001	0.4698
Black	0.973 (0.869, 1.090)	0.6373		1.089 (1.019, 1.165)	0.012	
Other	1.051 (0.917, 1.204)	0.4769		1.067 (1.020, 1.116)	0.0051	
Unknown	1.031 (0.921, 1.153)	0.5989		1.035 (1.003, 1.069)	0.032	
**CKD**						
NO	0.986 (0.926, 1.051)	0.6711	0.1803	1.052 (1.037, 1.068)	<0.0001	0.3753
Yes	0.915 (0.835, 1.003)	0.0572		1.030 (0.985, 1.077)	0.2	
**Hypertension**						
NO	0.982 (0.887, 1.088)	0.7331	0.6549	1.049 (1.024, 1.075)	<0.0001	0.9225
Yes	0.957 (0.901, 1.015)	0.1441		1.051 (1.032, 1.069)	<0.0001	
**Cerebral infarction**						
NO	0.975 (0.921, 1.033)	0.3866	0.3175	1.053 (1.036, 1.069)	<0.0001	0.4864
Yes	0.913 (0.813, 1.026)	0.1271		1.039 (1.006, 1.073)	0.0194	
**Myocardial infarction**						
NO	0.970 (0.913, 1.030)	0.3173	0.6651	1.052 (1.035, 1.069)	<0.0001	0.7254
Yes	0.947 (0.863, 1.039)	0.2494		1.045 (1.015, 1.077)	0.0032	
**Diabetes**						
NO	0.988 (0.930, 1.049)	0.6893	0.0979	1.054 (1.037, 1.071)	<0.0001	0.4023
Yes	0.899 (0.816, 0.991)	0.0326		1.039 (1.011, 1.068)	0.0056	
**Malignant cancer**						
NO	0.948 (0.898, 1.001)	0.0554	0.0687	1.054 (1.037, 1.071)	<0.0001	0.4023
Yes	1.107 (0.942, 1.301)	0.2161		1.039 (1.011, 1.068)	0.0056	
**GCS**						
3–8	0.984 (0.904, 1.071)	0.709	0.4366	1.035 (1.010, 1.061)	0.0062	0.0896
9–12	1.041 (0.930, 1.166)	0.4836		1.037 (1.011, 1.064)	0.0048	
13–15	0.952 (0.876, 1.035)	0.2493		1.071 (1.046, 1.097)	<0.0001	

Blood urea nitrogen-creatinine ratio, BUNCR; OR, odds ratio; CI, confidence interval; CKD, chronic kidney disease; GCS, Glasgow coma scale.

Adjusted for age, gender, race, chronic kidney disease, hypertension, cerebral infarction, myocardial infarction, diabetes, malignant cancer, Glasgow coma scale.

## 4 Discussion

In this study, the relationship between BUNCR and 30-day and 1-year mortality in patients with NTIH was determined using generalized linear models (GLM); more specifically, logistic regression and GAM. Fully adjusted GLM analysis demonstrated no statistically significant linear association between BUNCR and patient mortality. Furthermore, both the GAM and two-piecewise regression models revealed a non-linear relationship between BUNCR and 30-day/1-year mortality. To the best of our knowledge, this non-linear relationship has not been previously reported. Our study revealed that, to the left of the inflection point, a decrease of 1 unit in BUNCR corresponded to a 17.7% reduction in 30-day mortality rate and a 6.3% reduction in the 1-year mortality rate. Conversely, to the right of the inflection point, an increase of 1 unit in BUNCR was correlated with a 1.6% increase in the 30-day mortality rate and a 3.6% increase in the 1-year mortality rate.

Previous research has traditionally focused on using BUN or creatinine as independent indicators to predict their association with patients experiencing cerebral hemorrhage or stroke (26). However, our study represents the first attempt to analyze the association between BUNCR and mortality in patients with NTIH by using a novel approach that considers the ratio of the 2 markers. According to our findings, the relationship between BUNCR and patient mortality resembled a U-shaped curve, with mortality gradually decreasing on the left side of the inflection point as BUNCR increased. This observation considers the fact that, as the denominator of the ratio, creatinine levels tend to be higher in patients with better physical condition (27), indicating greater muscle mass compared to those who are physically weaker, thus resulting in a decrease in BUNCR. Previous studies have indicated that previous muscle mass plays a crucial role in the occurrence of postoperative complications in patients who experience cerebral hemorrhage, such as pulmonary infections and deep vein thrombosis in the lower limbs (28). For this group of patients, BUNCR can serve as a reliable basis for personalized nutritional supplementation and early rehabilitation training.

As previously described, BUN predominantly originates from the catabolism of endogenous proteins. For individuals severely affected by traumatic brain injury, cerebral metabolic demand surges by 40% compared with an uninjured brain, consequently precipitating a conspicuous surge in proteolytic activity (29). Previous investigations have posited that elevated intracranial pressure consequent to cerebral hemorrhage causes hypothalamus-pituitary axis dysregulation, culminating in the excessive release of antidiuretic hormones and renin-angiotensin, ultimately resulting in diminished renal perfusion, reduced glomerular filtration rate, and impaired urea nitrogen excretion (30). Furthermore, in cases in which the hemorrhage is located in close proximity to the midline and engenders substantial bleeding volumes, its propensity to involve the hypothalamus and incite stress-induced disturbances is intensified (31). Additionally, enzymatic degradation of hematoma tissue yields amino acids, which, upon entry into the systemic circulation, undergo reductive deamination, yielding ammonia. Subsequently, ammonia re-enters the circulation, contributing to urea biosynthesis and ultimately culminating in increased BUN concentrations (32). In our observations, we noted that urea nitrogen levels exhibited early elevations in the disease course, indicative of the initial pathophysiological cascades pertaining to brain injury, and the extent of urea nitrogen elevation was markedly more pronounced in patients prognosed with poorer outcomes than in counterparts with more favorable results. This finding aligns with those reported by Luo et al. (26). It is discernible from the foregoing that BUNCR mirrors not only the severity of cerebral injury but also patient muscular constitution, both of which have profound implications for NTIH prognosis. Therefore, when BUNCR resides within the nadir of the U-shaped curve, it signifies diminished cerebral injury and enhanced muscular mass, thereby indicating a more favorable clinical trajectory. In summary, we posit that BUNCR functions as an indirect conduit through which to understand the pathophysiological cascades following NTIH, and its dynamic oscillations mirror patient survival probabilities, thus endowing it with significant prognostic importance.

An accurate prognosis plays a crucial role in the diagnosis and treatment of NTIH because many critically ill patients with this condition may prematurely abandon aggressive therapy, resulting in unfavorable outcomes. Traditional prognostic indicators for NTIH include patient age, smoking and alcohol history, blood pressure, hematoma volume, coagulation function, and GCS score (33). Additionally, malignant tumors, blood glucose levels, and medical insurance coverage are vital factors that determine the long-term survival prospects of patients (34). In our study, we performed subgroup analyses and interaction tests on selected factors, revealing that the association between BUNCR and the prognostic mortality rate in patients with NTIH remains unaffected by confounding factors, establishing BUNCR as a stable prognostic predictor for NTIH. Compared with traditional prognostic markers, BUNCR is easily obtainable through routine blood tests and requires no additional costs or complex procedures. Moreover, BUNCR can reflect changes in kidney function, thus providing an indirect means of assessing patient metabolic status and the extent of kidney damage. By combining this ratio with other clinical indicators and imaging results, a more comprehensive evaluation of patient prognostic risk can be achieved, thereby supporting early intervention and proactive treatment.

The present study, however, had several limitations that should be acknowledged. First, BUNCR is influenced by multiple confounding factors, including common factors such as renal dysfunction and acute massive bleeding, leading to reduced blood volume, hemoconcentration status, and dietary protein intake. Although we excluded some of these confounding factors, the heterogeneity of patient conditions and various treatment requirements were not fully accounted for. Specifically, we did not statistically analyze the specific dosages and durations of hormones, sedatives, vasoactive drugs, or insulin used in the study. Furthermore, we did not conduct a baseline comparison of energy intake and nutritional pathways (total or partial enteral nutrition), which may introduce discrepancies in the research outcomes and potentially limit the generalizability of the BUNCR index. We believe that future studies should delve deeper into the impact of these factors to refine the prognostic utility of BUNCR in patients with NTIH.

Moreover, it is important to acknowledge that our data were sourced from a single institution, which may have introduced selection bias. The MIMIC-IV database contains clinical data from a single institution, which may not fully represent diverse populations and regions worldwide. Therefore, before applying these results to other populations, we recognize the need for further multicenter, large-sample, prospective studies to investigate the clinical utility of BUNCR in patients with NTIH. In light of these promising results based on BUNCR, we are also committed to monitoring this indicator in our clinical practice and will further substantiate this viewpoint as we gather additional clinical data.

## 5 Conclusion

In conclusion, this study focused on patients with NTIH and explored the association between BUNCR and mortality rates. Through innovative statistical analysis, we identified a U-shaped curve relationship that has not been previously reported. BUNCR has been confirmed to be a stable prognostic indicator reflecting the body’s metabolic status and muscle quality. These findings emphasize the importance of the early identification and proactive management of patients with adverse prognostic factors. Despite its limitations, this study highlights the potential clinical utility of BUNCR in guiding treatment decisions for patients with NTIH, thus contributing to better patient outcomes and reducing economic and health burdens.

## Data availability statement

The data analyzed in this study was obtained from the Medical Information Mart for Intensive Care III (MIMIC-III) database, the following licenses/restrictions apply: to access the files, users must be credentialed users, complete the required training (CITI Data or Specimens Only Research) and sign the data use agreement for the project. Requests to access these datasets should be directed to PhysioNet, https://physionet.org/, https://doi.org/10.13026/6mm1-ek67.

## Ethics statement

The requirement of ethical approval was waived by Medical Research Ethics Committee of the Second Affiliated Hospital, Nanchang University for the studies on humans because in this study, patients from outside the institution were included, and the MIMIC database is a publicly accessible and open database, which is exempt from ethical approval. The studies were conducted in accordance with the local legislation and institutional requirements. Written informed consent for participation was not required from the participants or the participants' legal guardians/next of kin in accordance with the national legislation and institutional requirements. The human samples used in this study were acquired from gifted from another research group.

## Author contributions

PC: Writing – original draft, Writing – review & editing, Formal Analysis. YJ: Writing – review & editing, Formal Analysis. JC: Writing – original draft, Writing – review & editing, Data curation. HF: Writing – review & editing, Data curation. JL: Writing – review & editing, Data curation. RY: Writing – review & editing, Data curation. HW: Writing – review & editing, Data curation. YW: Writing – review & editing, Data curation. SC: Writing – original draft, Writing – review & editing. YZ: Writing – review & editing, Conceptualization, Methodology.

## Footnotes

^1^ https://github.com/MIT-LCP/mimic-code/tree/main/mimic-iv
