# Supplementary material for: Prediction of prognosis in patients with nontraumatic intracranial hemorrhage using blood urea nitrogen-to-creatinine ratio on admission: a retrospective cohort study based on data from the medical information Mart for intensive care-IV database
Source: Front Neurol. 2024 Jan 5;14:1267815. doi: 10.3389/fneur.2023.1267815 (PMC10797125; doi:10.3389/fneur.2023.1267815)
Supplement: Supplementary file 1 [file Table_1.docx]

**Supplement Table 1.** In the basic model, the introduction of covariates and the exclusion of covariates in the full model were performed to observe the changes in the regression coefficient of BUNCR. Covariates that had an impact on the regression coefficient of BUNCR greater than 10% were subsequently removed from the analysis.

| **Covariate inspection and screening** | | | | |
| --- | --- | --- | --- | --- |
|  |  | **The regression coefficient** | |  |
|  |  | **Basic Model** | **Full Model** |  |
| **Covariate** | **+/- term** | **BUNCR** | **BUNCR** | **Selected** |
|  | **The initial regression coefficient.** | 0.0393 | -0.0101 |  |
| Age | Age | -0.0006 * | 0.0322 * | Yes |
| Gender | Factor (Gender) | 0.0424 | -0.0246 * | Yes |
| Race | Factor (Race) | 0.0421 | -0.0227 * | Yes |
| CKD | Factor (CKD) | 0.0456 * | -0.0107 | Yes |
| Hypertension | Factor (Hypertension) | 0.0391 | -0.0057 * | Yes |
| Cerebral infarction | Factor (Cerebral infarction) | 0.0394 | -0.0080 * | Yes |
| Myocardial infarction | Factor (Myocardial infarction) | 0.0362 | -0.0084 * | Yes |
| Diabetes | Factor (Diabetes) | 0.0412 | 0.0023 * | Yes |
| Malignant cancer | Factor (Malignant cancer) | 0.0395 | -0.0117 * | Yes |
| CHF | Factor (CHF) | 0.0407 | -0.0149 * | Yes |
| GCS | Factor (GCS) | 0.0301 * | -0.0143 * | Yes |
| HR | HR | 0.0391 | 0.0012 * | Yes |
| RR | RR | 0.038 | -0.0061 * | Yes |
| temperature | temperature | 0.0472 * | -0.0073 * | Yes |
| SBP | SBP | 0.0389 | -0.0119 * | Yes |
| SpO2 | SpO2 | 0.0395 | -0.0120 * | Yes |
| Lymphocytes | Lymphocytes | 0.0194 * | -0.0009 * | Yes |
| Monocytes | Monocytes | 0.0462 * | -0.0176 * | Yes |
| Neutrophils | Neutrophils | 0.0435 * | -0.0181 * | Yes |
| RBC | RBC | 0.0320 * | -0.0058 * | Yes |
| Platelet | Platelet | 0.0402 | -0.0231 * | Yes |
| CRP | CRP | 0.0376 | -0.0006 * | Yes |
| INR | INR | 0.039 | -0.0080 * | Yes |

Abbreviation: CKD, chronic kidney disease; CHF, congestive heart failure; GCS, Glasgow coma scale; HR, heart rate; RR, respiratory rate; SBP, systolic blood pressures; SpO2, Oxygen saturation; RBC, red blood cell; INR, international normalized ratio; CRP, C-reactive protein.
